# Online discussion for block teaching in postgraduate health professionals’ curriculum: the Ethiopian experience

**DOI:** 10.1186/1472-6920-14-29

**Published:** 2014-02-12

**Authors:** Bineyam Taye

**Affiliations:** 1 School of Medical Laboratory Sciences, College of Health Sciences, Addis Ababa University, Addis Ababa, Ethiopia; 2 College of Health Sciences, School of Public Health, Addis Ababa University, Addis Ababa, Ethiopia

**Keywords:** Online discussion, Block teaching, Ethiopia

## Abstract

**Background:**

Online discussions as a method of instruction are a new approach in Ethiopia. There is no previous study in the Ethiopian context that has assessed students’ engagement and learning experience using this instruction method, which may offer a valuable complement to other instruction methods for intensive block teaching in a resource-limited environment. The aim of this study was to assess the value of online discussions in supporting students’ engagement and interaction with their peers and teachers in a block teaching postgraduate health professionals’ curriculum.

**Methods:**

The research was conducted at Addis Ababa University College of Health Sciences, School of Medical Laboratory Sciences (SMLS), which has structured the curriculum around intensive block teaching. Between December 2011 and February 2012, two groups of full-time (N = 21) and part-time (N = 52) postgraduate students participated in online discussions as part of a Biostatistics and Research Methods module, in addition to other instructional methods. Every week, the course instructor initiated the online discussion by posting an assignment and articles with a few discussion questions. To evaluate the participants’ collective learning experience, the content of the email messages generated during these online discussions was analyzed qualitatively.

**Result:**

A total of 702 emails were exchanged during the three week module, of which 250 emails (35.6%) were posted by full-time students and 452 emails (64.4%) by part-time Continuing Education Program (CEP) students. During the online discussion forum, students identified different statistical data analysis tools and their application for given data sets. In terms of message contents, 67% of full-time and 64% of part-time students’ messages were classified as learning experiences. However, a slightly higher proportion of part-time students’ posts were social messages. The majority of students in both groups reported high levels of satisfaction with their online experience.

**Conclusion:**

Online discussion could be a valuable addition to face-to-face classroom teaching to improve students’ engagement and interaction in an intensive block teaching postgraduate curriculum where learners are engaged in a full work load with academic studies.

## Background

Graduate education in Ethiopia, as in many parts of Africa, is a relatively recent phenomenon. At Addis Ababa University (AAU), graduate study was launched in 1978 with eight MSc programs, while PhD training started in 1987. One major factor that prompted the university to launch graduate study programs was the need to provide qualified academic staff for higher education institutions in the country. As part of achieving this aim, AAU initiated Business Process Reengineering (BPR) and restructured the existing semiannual (semester-based) mode of course delivery to intensive block teaching that aimed to engage the learner in a full work load in the form of self-directed study (such as reading, research, lab work, practicum, etc.), as well as interactive and collaborative study [1]. Intensive block teaching is a restructuring of the school day into classes much longer than the traditional 50-minute period. In one common form, students have four long class periods per day instead of seven or eight. A course that normally covered the entire school year could then be compressed into an intense half-year course [1-3]. Proponents of block teaching claim that it reduces fragmentation of instruction, accommodates more effective teaching practices, and expands opportunities for individualized instruction. Critics, on the other hand, maintain that instructional time over the school year is actually reduced, content coverage is curtailed and superficial, and therefore it may be difficult to justify the amount of time and effort required by both teachers and students to implement the limited course content during a block week of study [4]. Furthermore, students’ interactions with their peers and teachers may be hampered due to full engagement in academic studies to complete the module within a short block schedule. To address this concern, alternative strategies that can increase students’ interactivity with their peers and teachers should be considered, and online web based teaching could be a valuable complement to more established teaching methods.

Online education has expanded opportunities for flexible, convenient and interactive methods of instruction. This includes opportunities for sharing ideas, posing questions, and presenting individual discoveries as they occur and at the student’s convenience. Interactivity here develops outside official teaching hours and learning programs. It challenges students and makes them more independent [5,6]. Moreover, online discussions can be an excellent pedagogical tool for teaching and practicing critical thinking beyond time and place constraints of the physical classroom, thus providing a stimulating supplement to standard teaching practices [7].

As online methods of instruction are new in Ethiopia, no previous study has tried to assess students’ engagement and learning experience using these methods. This study attempted to assess the value of online discussions in supporting students’ engagement and interaction with their peers and teachers, and to identify areas of major collective learning using statistical tools for data processing and analysis.

## Methods

### Study setting and context

A descriptive study was conducted at Addis Ababa University (AAU) College of Health Sciences, School of Medical Laboratory Sciences (SMLS). The school, in response to AAU reforms, has restructured the postgraduate curriculum, and modules are delivered in a block teaching format. Each block is 4 weeks for full-time - and 8 weeks for part-time postgraduate students, respectively. In the December 2012 academic program, new postgraduate students in both the regular full-time and part-time education programs were requested to participate in online discussions via a Google group created by the instructor. Online discussions as part of a 'Mentoring and Learning Web’ (ML Web) has been reported earlier by the Regional Institute Fellows of the Foundation for Advancement of International Medical Education and Research (FAIMER) in Philadelphia, USA [8]. The investigator is a 2011 FAIMER Institute fellow, and as part of this fellowship, had training and experience in facilitation of ML Web online discussion.

### Study population

Two groups of postgraduate students were included in the study: the first group was full-time postgraduate students attending the program in weekday daytime hours (8 a.m.-5 p.m.) and exempted from their routine work. The second group was part-time postgraduate students registered in the continuing education program (CEP) and attending the module in their spare time, such as evening (6 p.m.-8 p.m.) and weekend daytime hours (8 a.m.-5 p.m.) while employed full-time in different organizations, such as government and private hospitals, private medical colleges and non-governmental organizations (NGO). A total of 21 regular full-time and 52 part-time postgraduate students were included in the study.

### Online web discussion process

The first module of the postgraduate program, Biostatistics and Research Methods, was selected for assessment of online web discussion. The group discussion was based on adult learning principles in which the learner is motivated by problem-centred or task-centred education, which should consist of realistic scenarios that they may anticipate encountering [9]. The module was facilitated in December 2011 for full-time students and January to February 2012 for part-time postgraduate students. The main subject of the discussion was how to use statistical tools for a given set of research data. The discussion themes were organized under descriptive and inferential statistics. The discussion was carried out parallel to the routine classroom teaching and moderated by the course instructor. Every week, the course instructor posted an assignment and articles with a few discussion questions. These instructor posts were designed to parallel the topics covered in routine class room teaching. Students were encouraged to reflect and express their free and spontaneous responses, raise questions, and share their problems and experiences. The role of course instructor was to monitor and facilitate the discussion and ensure prompt feedback to student posts. Students’ satisfaction toward their online learning experiences was assessed using an online survey questionnaire through SurveyMonkey. SurveyMonkey is an online service that allows users to create web-based surveys. This service offers both a free account and a paid account, which includes enhanced features [10].

### Categories of messages

Students’ messages in the online discussion group were divided into two categories:

Social presence messages include comments that contributed to social interaction, but were not directly related to discussion topics.

Learning experience messages include comments directly related to discussion topics, such as summaries, clarifying queries, and answers to the posted question, which are important in facilitating and stimulating learning among participants.

### Ethical approval

Approval for the study was given by the Department Ethical Review Committee (DERC), School of Medical Laboratory Sciences, Addis Ababa University. The DERC approved use of oral informed consent documented by a witness after the objectives of the study had been explained. All subjects provided informed consent.

### Data analysis procedures

After consent was obtained from students, the content of emails generated during online group discussions were retrieved and sorted by date and time of posting. Based on the factors that affect the quality of online discussions as identified in the literature [11-13], coding schemes for social presence and learning experience comments were developed. Following the iterative process of categorizing, re-categorizing, and creating codes using actual comments in the transcript, a total of four social presence codes and seven learning experience codes were developed (Table 1) [12,14]. Using the existing transcripts of online discussions, content analysis was conducted based on a coding scheme to characterize the students’ learning experiences [15]. The coded data were entered into an Excel spreadsheet and then imported into and analyzed by SPSS (Statistical Package for the Social Sciences, Version 15.0) to generate the proportion of students’ messages by code type and group type (full-time or part-time).

**Table 1:** Coding of social and learning experience messages

**Social presence**	**Code**	**Learning experience**	**Code**
Greeting	S1	Direct response to discussion question	LE1
Expressing of wish	S2	Sharing learning material	LE2
Administrative information sharing	S3	Summarizing discussions	LE3
Praise, appreciation	S4	Asking questions	LE4
		Clarifying misconceptions	LE5
		Agreeing or disagreeing with other students	LE6
		Highlighting teaching points	LE7

## Results

A total of 702 emails were exchanged during the three weeks of block teaching, of which 250 emails (35.6%) were sent by full-time students and 452 (64.4%) were sent by part-time (CEP) students. Of these messages, 83.3% of full-time and 65.5% of CEP students’ emails were categorized as learning experience emails (Defined as a direct response to a discussion question, sharing learning material, asking questions, clarifying misconceptions, agreeing or disagreeing with other students, or highlighting a teaching point). Table 2 shows the analysis of participation during the three-week block teaching program. On average, 12 emails from full-time and 21 emails from part-time postgraduate students were exchanged each day respectively.

**Table 2:** Analysis of number of emails sent during the three week Biostatistics and Research design module at Addis Ababa University College of Health Science, School of Medical Laboratory Science

**Variable**	**Participant in online discussion**	
	**Full-time students (N = 21)**	**Part-time (CEP) students (N = 52)**
Total emails/(posting), n (%)	250 (35.6)	452 (64.4)
Learning experience emails, n(%)	208 (83.2)	296 (65.5)
Social emails n (%)	42 (16.8)	156 (34.5)
Average emails per day n (%)	11.9	21.5
Average emails per student during the module period n (%)	11.3	8.7

### Frequency of social and learning experience messages by contents

The frequency of social and learning experience messages was further analyzed using the predetermined coding scheme as indicated in Table 1. 67% of full-time and 64% of part-time students’ messages were classified as learning experience. Of learning experience messages, 45.4% of full-time and 41.9% of part-time students’ comments were direct responses to a discussion questions. 19.5% of CEP students’ comments asked questions of their peers or instructors, and 15.1% of full-time students’ comments focused on clarifying misconceptions during the three weeks of online discussion. On the other hand, 33% of full-time and 36% of CEP students’ messages were classified as social presence messages (Table 3).

**Table 3:** Distribution of messages contents by code during the three weeks Biostatistics and Research design module at Addis Ababa University College of Health Science, School of Medical Laboratory Science

***Categories***	***Full-time***	***Part-time***
**Social presence N (%)**^**1**^	112 (33)	347 (36)
Greeting	21 (18.75)	87 (25.07)
Expressing of wish	28 (25)	69 (19.88)
Administrative information sharing	21 (18.75)	121 (34.87)
Praise, appreciation	42 (37.5)	70 (20.17)
**Learning experience N (%)**^**2**^	231 (67)	620 (64)
Direct response to discussion question	105 (45.4)	260 (41.94)
Sharing of learning material	14 (6.05)	87 (14.03)
Asking questions	35 (15.12)	121 (19.52)
Clarifying misconceptions	35 (15.12)	60 (9.68)
Agreeing or disagreeing with other students	14 (6.05)	40 (6.45)
Highlighting teaching points	28 (12.16)	52 (8.38)

^1^Total social presence messages and its proportion in each group.

^2^Total learning experience messages and its proportion in each group.

### Qualitative analysis of areas of students’ collective learning experience on different types of statistical tools

The categories of discussion on statistical tools for data processing and analysis under the heading descriptive and inferential statistics was presented as a simple non-hierarchical typology which represents conceptions of the phenomenon by the students and considered areas of collective learning experiences.

### Descriptive statistics theme

During the week dedicated to the topic of descriptive statistics, two categories of themes emerged from the analysis of the online discussion. The first category was best practices of data presentation; both groups posted comments such as, “*Tables or graphs are more effective data presentation scheme for nominal and ordinal data.”* The second category was proper utilization of measures of central tendency and dispersions. Most students noted that *“mean should be used for interval and ratio data with symmetric distribution, median and quartiles are used for ordinal, interval and ratio data whose distribution are skewed,”* and mode was mentioned as appropriate measure of central tendency for nominal data. Both full-time and CEP students also described the use of measures of dispersion such as inter-quartile range (IQR) to be used with the median and standard deviation (SD) with the mean.

### Inferential statistics theme

The theme that emerged during the week dedicated to inferential statistics was the selection of an appropriate statistical test for any given data set. Parametric tests were described as “*the most powerful statistical tests used to detect a difference if one exists and if the assumptions of about the data met i.e. the collected data should be on an interval or ratio scale and described using the mean and standard deviation (SD).”* T tests, ANOVA, correlation, and regression were the discussion areas. It was also noted by both groups that “*non parameter statistical tests are more robust and do not make any assumptions about the underlying distribution of the data”.* The Wilcoxon signed-rank test, Mann–Whitney U test and Spearman’s rank correlation coefficient were among the non-parametric tests discussed. TheChi square test was described as important method of statistical test for nominal and ordinal (categorical) variables.

### Students’ online reflection when they faced challenges during discussion and exercise

Students expressed their difficulties and challenges during the online discussion in different ways. Sample student posts include: “*I am confused with analysis of rows and columns (like 2x3) tables*” (Respondent 19), *“Can we compute odds ratio/relative risk for these kind of tables?”* (Respondent 26), *“Can you guys help me, I can’t down load SPSS data?”* (Respondent 7), *“I am not clear with the assignment!” *(Respondent 11). Students and course instructors responded immediately to alleviate the problems and to continue the discussion. Additionally, students expressed their impressions of the discussion and assignments. One student said that *“I am telling you frankly that I have enjoyed the discussion very well, it strengthen my knowledge about probability”* (Respondent 4).

### Students’ feedback on online discussion

An online satisfaction survey was administrated to assess students’ satisfaction toward their online experience. About 70% (51/73) of students returned the satisfaction survey. Of those who responded, two third (66%) reported that they were extremely satisfied on the content of the module; 64% reported being extremely satisfied with online discussion. None of the students in either group expressed dissatisfaction on the evaluation variable regarding the online session (Table 4). One student expressed that “*the online discussion methods is the first experience for almost of us and I found it as Interactive*, *student-centered*, *and helped to familiarize to the technology.”* (Respondent 18).

**Table 4:** Students’ feedback about the three-weeks- online discussion

**Evaluation parameter**	**Extremely satisfied**		**Moderately satisfied**		**Slightly satisfied**		**Neither satisfied nor dissatisfied**		**Dissatisfied**	
	**N**	**%**	**N**	**%**	**N**	**%**	**N**	**%**	**N**	**%**
Were you satisfied with the course content?	34	66.7	17	33.3	0	0	0	0	0	0
How reasonable are the expectations for student achievement at course?	11	22.4	32	65.3	6	12.2	0	0	0	0
How organized was the course content?	29	52.9	17	37.3	5	9.8	0	0	0	0
How reasonable are the sessions explain the objectives of the course clearly?	19	38.8	27	55.1	3	6.1	0	0	0	0
Are you satisfied with the online teaching experience?	33	64.7	17	33.3	1	2	0	0	0	0

## Discussion

This study is the first, to my knowledge, to assess students learning experience using online discussion as a complementary method of instruction in a block teaching curriculum in Ethiopia, where resources and access to internet are limited. Despite this technical challenge, students sent, on average, 12 emails from full-time and 21 emails from part-time postgraduate students per day. In terms of message contents, 67% of full-time and 64% of part-time students’ messages were classified as learning experience messages. This finding is in contrast with the study conducted by Kim et al., where 54% of comments by the students were social messages [16]. It was hypothesized that the higher proportion of student comments focused only in the discussion topic in this study was due to the fact that the online discussion format was designed to supplement the existing classroom teaching and monitored by a course instructor parallel to the routine classroom teaching.

Part-time postgraduate students were found to have more social postings than full-time students. This pattern could be due to the increased time constraints of these students, who must be physically present in the school compound to exchange information during regular working hours while also working full-time in different organizations.

One major area of discussion was best practices of statistical data processing and analysis. Similar evidence of emerging areas of online discussion was documented in study by Dongre et al. indicating the possibility of online discussion in learning statistical tools [17].

Some students used the online discussion to express confusion or ask questions about particular topics. This type of discussion was also noted in a study by Woo et al., which indicated that web-based methods of instruction enhance and stimulate learning and encourage learners to seek out answers or help as soon as the need arises. This function of online discussion could be a valuable supplement to face-to face discussion by giving “voice” to silent students, thereby providing a more egalitarian learning environment [18]. Moreover, online discussion increases students’ involvement with the course content outside the regular classes, as online discussions leave transcripts available for review and feedback [19,20].

The majority of students reported high satisfaction with their online experience, which is consistent with studies in other settings [21,22]. Students’ open-ended comments about the online discussion were mainly positive regarding convenience, interaction and reflection. The opportunity to exercise choice and flexibility in learning was among the identified reasons that students wanted to participate on online discussion throughout the module weeks. Despite students’ positive feedback toward their online discussion experiences, it should be noted that online learning has several potential weaknesses: its impersonal approach, lack of spontaneous response compared to classroom discussions, the risk of overloading students with information and links, and the need for special equipment and skills and access to internet services [23].

### Limitation of the study

There are several limitations to this study. First, generalizability of the findings may be limited by the sample population. Specifically, only two groups of postgraduate students were involved, and the study was conducted at a single center. Second, since this online discussion forum was established as part of the requirement for completion of the course, it was not possible to determine whether the observed increased frequency of students’ participation in online discussion resulted from self-motivation or effort to meet the course requirements. Third, online discussion forums as a method of instruction for block teaching is a relatively new technique, and more controlled studies with a larger group of students engaging in online block teaching should be considered to explore possible issues related to instructional format.

## Conclusions

Blended use of online discussion and face-to-face classroom teaching supports students’ engagement and interaction with their peers and teachers, especially in the absence of enough qualified staff to provide guidance for students individually. More importantly, it is a valuable addition to an intensive block teaching postgraduate curriculum where learners are engaged in full work load with academic exercises that may limit interaction with their peer and teachers.

## Competing interests

The author declares that there is no conflict of interest associated with the publication of this manuscript.

## Authors’ contributions

BT conceived and designed the study, collected data, performed data analysis and drafting the manuscript and critically reviewed the manuscript.

## Pre-publication history

The pre-publication history for this paper can be accessed here:

http://www.biomedcentral.com/1472-6920/14/29/prepub
